# A very rare case of a thoracobrachialis muscle

**DOI:** 10.1007/s00276-023-03240-6

**Published:** 2023-09-21

**Authors:** Nicol Zielinska, Piotr Karauda, Andrzej Węgiel, Bartłomiej Szewczyk, Łukasz Olewnik

**Affiliations:** Department of Anatomical Dissection and Donation, Medical University of Lodz, Lodz, Poland

**Keywords:** Accessory muscle, Biceps brachii, Chondroepicondylaris, Chondroepitrochlearis, Pectoralis quartus, Medial epicondyle, Neurovascular compression, Pectoralis major, Pectoroepicondylaris, Thoracobrachialis

## Abstract

The anterior arm compartment includes the biceps brachii muscle, brachialis muscle, and coracobrachialis muscle, and they are characterized by different morphological variations. During standard anatomical dissection of right upper limb, an variant muscle was found. It originated from the fascia covering the long head of biceps brachii and from the tendon of pectoralis major muscle. It also connected to the brachial fascia. It also demonstrated a long thin tendon which was distally attached to the medial epicondyle of humerus. Knowledge about the morphological variations in this region is clinically important because of their direct correlation with neurovascular structures, which may lead to pathologies such as tingling, muscle weakness, paresthesia, and loss of sensation.

## Introduction

The anterior arm compartment includes the biceps brachii muscle, brachialis muscle, and coracobrachialis muscle, and they are characterized by different morphological variations, including additional muscle bellies and tendons. The locations of their proximal and distal attachments may also vary, as may the nature of their innervation, vascularity, or course of these neurovascular structures relative to anterior arm compartment muscles [1]. Also, as muscles from the pectoralis region or back muscles have their insertions on the humeral bone, their different morphological variants may be observed during standard anatomical dissection of upper limb and surrounding regions [1].

Some accessory structures may also occur, like the coracobrachialis longus muscle, coracobrachialis superior muscle, axillary arch muscle, pectoralis quartus muscle, pectoralis tertius thoracoepicondylaris and thoracobrachialis [1, 7]. Accessory structures may be associated with some pathologies causing neurovascular compression. These usually cause entrapment of brachial plexus structures [14].

During standard anatomical dissection of the right upper limb, an variant muscle was found. It originated from the fascia covering the long head of biceps brachii and from the pectoralis major muscle tendon, from where it connected with the brachial fascia. A long thin tendon was distally attached to the medial epicondyle of the humerus Knowledge of the morphological variations in this region is clinically important because of their direct correlation with neurovascular structures, which may give rise to certain pathologies and symptoms.

## Case report

A 91-year-old at death male cadaver donated to science was subjected to routine anatomical dissection for research and teaching purposes at the Department of Anatomical Dissection and Donation, Medical University of Lodz, Poland. The body had been donated to science. The region of the right upper limb was subjected to traditional anatomical dissection [7, 8], revealing the presence of a morphological variation in the region.

An variant muscle in the anterior compartment of right upper limb was found. It originated from the tendon of the long head of the biceps brachii. At this point, it was 7.40 mm wide and 0.97 mm thick. Its proximal origin was from the tendon of the pectoralis major muscle, and the distance between these two attachments was 46.03 mm.

**Fig. 1:**
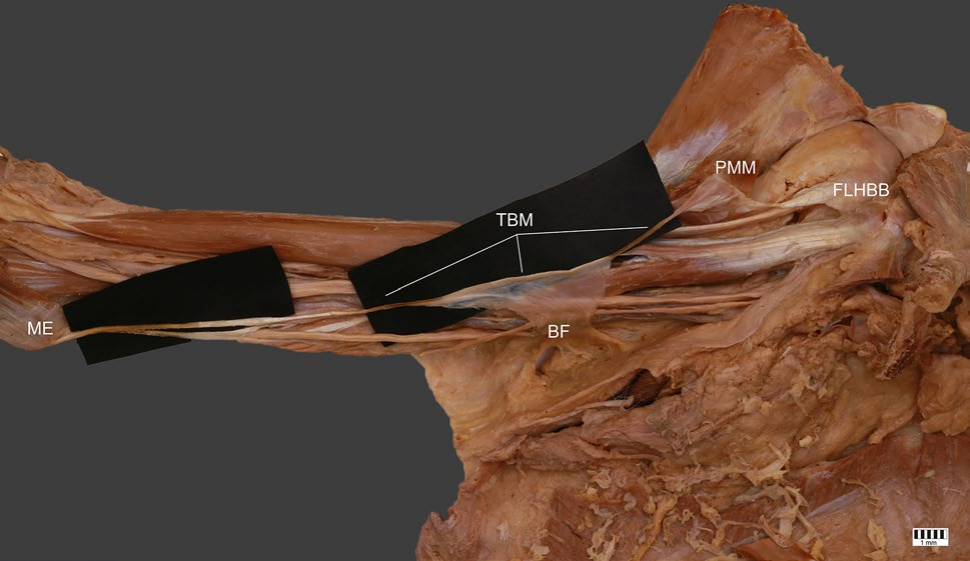
Thoracobrachialis muscle. *ME* medial epicondyle, *TBM* thoracobrachialis muscle, *PMM* pectoralis major muscle, *FHLBB* fascia of long head of biceps brachii, *BF* brachialis fascia

**Fig. 2:**
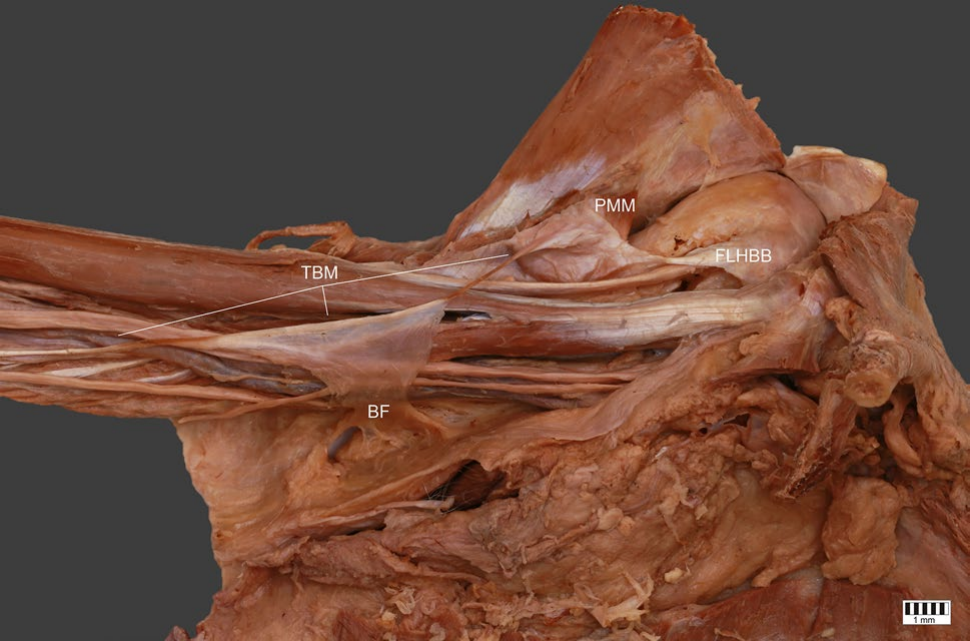
Thoracobrachialis muscle. *ME* medial epicondyle, *TBM* thoracobrachialis muscle, *PMM* pectoralis major muscle, *FHLBB* fascia of long head of biceps brachii, *BF* brachialis fascia

**Fig. 3:**
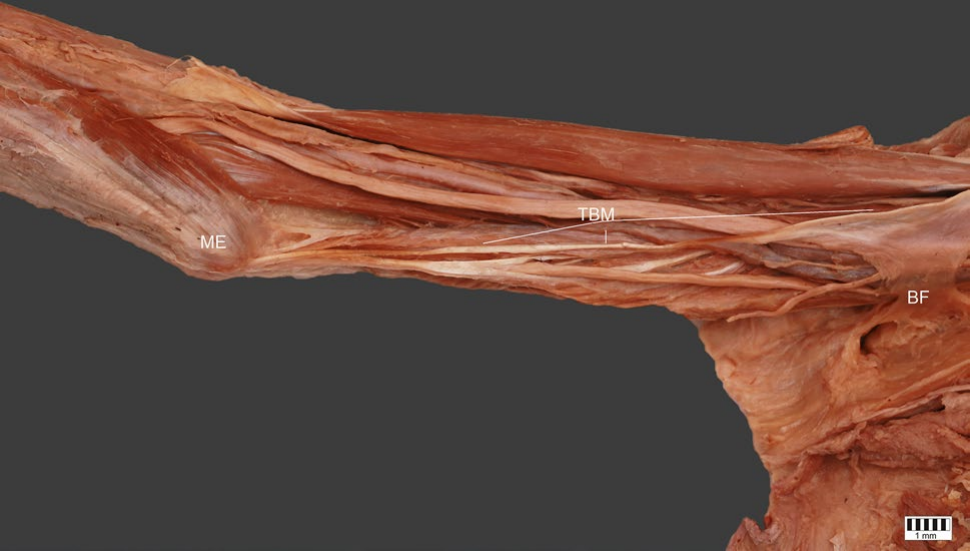
Thoracobrachialis muscle. *ME* medial epicondyle, *TBM* thoracobrachialis muscle, *PMM* pectoralis major muscle, *FHLBB* fascia of long head of biceps brachii, *BF* brachialis fascia

The structure then moved down and was connected with the brachial fascia. After this connection, a long thin tendon 121.08 mm in length was observed; this was proximally 2.17 mm wide, and 0.34 mm wide. This part was inserted to the medial epicondyle of the humerus. The width of insertion was 6.33 mm, and thickness 0.87 mm—Figs. 1, 2, 3.

The total length of all structures, i.e. from the upper part of the attachment to the pectoralis major to its attachment to the medial epicondyle of the humerus, was 216.54 mm.

The measurements were collected using an electronic caliper (Mitutoyo Corporation, Kawasaki-shi, Kanagawa, Japan). Each measurement was repeated two times with an accuracy of up to 0.01 mm.

No other morphological variabilities were found while dissecting the upper limb.

## Discussion

As mentioned above, during anatomical dissection of the right upper limb, a variant muscle was found. It originated from the fascia covering the long head of the biceps brachii, and from the tendon of the pectoralis major muscle. After that, it was also connected with the brachial fascia. Following this, a long thin tendon was distally attached to the medial epicondyle of the humerus.

This accessory structure appears to be a variant structure, as is does not completely match any descriptions given in the previous literature.

The closest match is the chondroepitrochlearis muscle, however, most reports indicate that it originates from the costal part of the ribs, or their cartilages [4]. This anomalous muscle was firstly described by Duvernoy (who named it) in 1855, as cited by Wood [13]. In the present case, no such proximal attachment was observed. However, it is possible that the identified structure may be an anatomical variation of the chondroepitrochlearis muscle.

This variant structure has been recorded many times. For example, Testut [11] described it as a structure originating from the 5th and 6th costal cartilages, going towards the axillary region, and dividing into two bundles: the upper one inserted on the anterior border of the humerus at the level of the tendon of the pectoralis major muscle, and the lower one, connected with the brachial aponeurosis, and attached to the medial epicondyle, just above common mass of the muscles inserted to the medial epicondyle. Another supernumerary bundle, arising from the abdominal aponeurosis and 6th costal cartilage was also observed. Its distal attachment was located on the medial epicondyle [11].

This variation has been also observed by Sommering, by Struthers, by Caldani, by Theile, by Cruveiller, by Develie. Using their observations and also the more recent cases, the chondroepitrochlearis muscle may be described as a structure coursing along the lower border of the pectoralis major muscle. It can originate from the 4th, 5th, 6th ribs (or its cartilages) or from the abdominal aponeurosis. On reaching the humerus, it throws itself on a tendon attached to the medial epicondyle, and it may be also connected with the brachial aponeurosis and the internal intermuscular septum [11].

Testut [11] also summarized variations of the chondroepitrochlearis muscle. The first one represented by few bundles from the pectoralis major muscle or from a supernumerary muscle. The second variation characterized by tendon originating from the capsule of the shoulder joint. The third type may be described as a structure originating from the serratus anterior muscle. The fourth variation of the chondroepitrochlearis muscle is represented by tendon receiving a muscle bundle from the pectoralis major muscle (in its middle part). The fifth variant was described as a tendon attached to the medial epicondyle originating as a double bundle (the first one from the pectoralis major muscle, and the second one from the latissimus dorsi muscle) [11].

Also Di Gennaro [2] found it to originate from the pectoral region and insert to the medial epicondyle. In turn, Redler et al. [10] describe a structure originating from the pectoralis major muscle and attaching distally to the coracobrachialis fascia, brachial fascia, and medial intermuscular septum; despite it not having any attachment to the ribs or medial epicondyle, it was also classified as a chondroepitrochlearis muscle [10]. Trobs et al. [12] also noticed something similar, but an insertion was located on the medial epicondyle.

Over the years, many variations of this muscle have been named. For example, the pectoroepicondylaris muscle [4] originates as a bilaminar tendon from a deep layer of the pectoralis major muscle, and inserts to the medial intermuscular septum and medial epicondyle. Additionally, the chondrofascialis muscle originates from the costochondral junction of fifth rib and attaches to the medial fascia of the arm, with an insertion into the humerus medial intermuscular septum [1].

Lama et al. [5] identified a muscle originating from the inferior aspect of the pectoralis major muscle and inserting to the fascia covering biceps brachii and the lateral lip of the intertubercular sulcus. Although its origin is not located on ribs, it was labelled the chondrohumeralis (or chondrobrachialis) muscle [5]. Another variation was the costohumeral muscle, originating from the sixth rib near the costochondral junction, inserting to the medial epicondyle [3].

Another muscular variation in this region is the thoracoepicondylaris muscle, as proposed by Loukas et al. [6]. This name would later encompass all muscular variations originating from the pectoralis major muscle, or latissimus dorsi muscle, or costal cartilage and inserting to the medial epicondyle.

Considering the diversity of these variations, their names and the close proximity of their attachments, it is difficult to classify the muscular variants in this region, Suresh et al. [9] proposed the term thoracobrachialis muscle a new name for all variants. This new classification included four types of the thoracobrachialis muscle based on its course and insertion (part of the brachial region). Type I was represented by origin located in the pectoral region and distal attachment to the intertubercular sulcus, or to the fascia covering the biceps brachii muscle or coracobrachialis muscle, or to the latissimus dorsi muscle. A common feature for this type was an insertion on the proximal third of the arm. Type II was characterized by muscular slip/tendon inserted to the middle third of the arm, for example: intermuscular septum, or fascia over the biceps brachii muscle or coracobrachialis muscle. Type III was represented by insertion to the distal third of the arm, for example the intermuscular septum or medial epicondyle. Finally, Type IV included other unusual presentations of this region, such as the pectoepicondylaris muscle [9].

During the present anatomical dissection, an interesting case was found. The muscular slip was arising from the fourth to sixth ribs, from where it passed under the lower border of the pectoralis major muscle. It then passed through the axillary region attached to the biceps brachii muscle fascia. Following this, this variant muscle was represented by a tendinous structure attached to the anterior compartment of the arm, and finally it inserted into the medial epicondyle.

One key difficulty in classifying our present case is that although, some known muscular variations originate only from the pectoralis major muscle (not from costal cartilages) and attach to the medial epicondyle, none demonstrate any additional proximal attachment from the fascia covering the biceps brachii. As such we propose that this new structure be classified as Type IV according to Suresh et al. [9].

An understanding of all muscular variations, especially accessory structures, is especially important when dealing with neurovascular compression. The branches of the brachial plexus, for example median nerve or ulnar nerve, may be entrapped, resulting in loss of function of innervated muscles or paresthesia and sensory disturbances of innervated skin regions. However, in addition to the neural structure, the axillary or brachial vessels may also be compressed, which may be observed as a thrombosis, change of skin color or edema.

In this case, described variant muscle is close to the median nerve and brachial artery. This may result in compression of median nerve, potentially resulting in the impairment of function of the anterior compartment of forearm, and weakness and numbness in the radial three and a half fingers. Brachial artery compression may result in pain, numbness, tingling, coldness, and weakness of the affected upper extremity. Therefore, the patient reports such symptoms, it is recommended to consider the presence of additional structures within the chest and upper limb muscles, including the thoracobrachialis muscle.

## Conclusion

Due to the substantial number of the muscular variations observed in pectoral region characterized by a distal attachment located on the upper limb and different names proposed by various authors, the classification of a new variation can sometimes be problematic. Variant structures associated with this region can cause some types of neurovascular compression, and therefore, knowledge about them may be helpful during diagnosis of patents reporting loss of muscle function, tingling, paresthesia, or sensory loss in upper limb regions.

## Data Availability

Please contact authors for data requests (Łukasz Olewnik PhD—email address: lukasz.olewnik@umed.lodz.pl).
